# A retrospective study on atrial fibrillation after coronary artery bypass grafting surgery at The National Heart Institute, Kuala Lumpur

**DOI:** 10.12688/f1000research.13244.2

**Published:** 2018-08-01

**Authors:** Ahmad Farouk Musa, Chou Zhao Quan, Low Zheng Xin, Trived Soni, Jeswant Dillon, Yuen Kah Hay, Rusli Bin Nordin

**Affiliations:** 1 School of Medicine and Health Sciences, Monash University Malaysia, Subang Jaya, Malaysia; 2 Department of Cardiothoracic Surgery, National Heart Institute, Kuala Lumpur, Malaysia; 3 School of Pharmaceutical Sciences, Universiti Sains Malaysia, Penang, Malaysia

**Keywords:** atrial fibrillation, coronary artery bypass grafting, incidence, predictors, outcomes

## Abstract

**Background: **Atrial fibrillation (AF) is common after cardiac surgery and has been associated with poor outcome and increased resource utilization. The main objective of this study is to determine the incidence of POAF in Malaysia and identify the predictors of developing POAF. The secondary outcome of this study would be to investigate the difference in mortality and morbidity rates and the duration of intensive care unit (ICU), high dependency unit (HDU) and hospital stay between the two.

**Methods: **This is a retrospective single-center, cross sectional study conducted at the National Heart Institute, Malaysia. Medical records of 637 who underwent coronary artery bypass grafting (CABG) surgery in 2015 were accrued. Pre-operative, operative and post-operative information were subsequently collected on a pre-formulated data collection sheet. Data were then analyzed using IBM SPSS v23.

**Results: **The incidence of POAF in our study stands at 28.7% with a mean onset of 45±33 hours post operatively. Variables with independent association with POAF include advancing age, Indian population, history of chronic kidney disease, left ventricular ejection fraction and beta-blocker treatment. The mortality rate is significantly higher statistically ( *p* < 0.05), and similarly the incidence of stroke. The incidence of other post-operative complications was also significantly higher statistically. The duration of ICU, HDU and hospital stays were statistically longer ( *p* < 0.001) with higher rates of ICU readmissions and reintubations seen.

**Conclusion: **We conclude that the incidence of POAF in Malaysia is comparable to the figures in Western countries, making POAF one of the most commonly encountered condition after CABG with similar higher rates of mortality, poor outcomes and longer duration of stay, and therefore increased cost of care. Strategies to reduce the incidence of AF after cardiac surgery should favorably affect surgical outcomes and reduce utilization of resources and thus lower cost of care.

## Introduction

Atrial fibrillation (AF) is the most common arrhythmia after cardiac surgery with a reported incidence of 15–40%, a significant leap in incidence compared to the normal population ^1^. Patients who underwent valve surgery or combined valve and coronary artery bypass grafting (CABG) have higher incidence of postoperative AF (POAF) than patients having CABG alone ^2^. AF is especially common after mitral valve surgery, occurring in as many as 64% of patients ^3^. POAF is self-limiting in most cases, but even when it is unlimited, it requires additional medical treatment and a prolonged hospital stay, and it consequently increases the cost of operative treatment ^4– 6^.

POAF is generally associated with an increased risk of morbid consequences and even mortality. The risk of developing cerebral infarction is more than doubled in patients with POAF, with a reported incidence of 5.3% compared to patients without POAF, which has a reported an incidence of 2.4% ^7^. Stroke remains the main morbid event for POAF, as the recurrence rate for stroke stands at 20% per year regardless of whether it is chronic or intermittent AF. Other complications such as post-operative congestive heart failure, renal failure, infection and neurological changes all experienced significant increases in patients with POAF ^2, 7^.

POAF is also noted to increase the risk of mortality. A two-fold increase is reported in both the short-term mortality rate (1.9% to 3.6%, *p*<0.001) as well as the 1-year mortality rate (3.4% to 6.9%, *p*<0.001) in patients with POAF compared to their healthy counterparts. A similar pattern can also be seen in the 4-year mortality rate (8.8% to 15%) and the 8 year mortality rate (17% to 29%) ^8^. In addition, patients with POAF are reported to be at a higher risk of dying from embolism ^9^.

The duration of hospital stay of POAF patients is extended as well (10 days in POAF group *vs* 7 days in non-POAF group) ^9, 10^. Furthermore, patients with POAF are also more likely to be re-intubated and readmitted ^11^.

### Risk factors

***Co-morbid history.*** Several risk factors have been identified to increase a patient’s possibility of developing POAF ^12^. Advanced age has consistently been related to a higher incidence of POAF. Aging has been associated with remodelling of the atria ^13^.

Another pre-operative risk factor that has been consistently significant is cardiac remodelling in the form of left atrial dilatation and left ventricular hypertrophy, which may result in diastolic dysfunction ^14^. These risk factors may be related to each other as advanced aged is associated with degenerative and inflammatory changes in the cardiac anatomy. These anatomic changes provide triggers for an episode of POAF ^15^. On top of that, underlying cardiac disease, such as hypertension and valvular abnormalities, which are more common in the elderly, can cause cardiac remodelling as well. These two are also independent predictors of POAF ^16^.

Male gender has inconsistently been associated with POAF. Sex differences in ion-channel expression and hormonal effects on autonomic tone may explain this disparity ^17^. Recently, metabolic syndrome (MS) as a risk factor to POAF has been brought to the attention of clinicians. Echahidi *et al* found a significant increase in the incidence of POAF in patients with mild as well as moderate-severe obesity ^18^. On top of that, pre-operative low-density lipoprotein cholesterol (LDL-C) was found to be significantly higher in patients who developed POAF, enhancing the predictive value of a pre-operative lipid profile ^19^.

Other pre-operative risk factors include chronic renal failure, previous history of AF and a history of rheumatic heart disease ^20^. Hypothyroidism has been linked to an increased incidence of POAF in a recent small retrospective review ^21^. It remains to be seen whether this linkage is reproducible in future large cohort studies but this finding is exciting as hypothyroidism is an easily treatable condition.

***Intra-operative.*** Valve surgery is the most consistently linked risk factor to POAF after cardiac surgery. Several authors have expressed the possibility that the anatomical changes in the atrium secondary to valvular disease are more important than the procedure itself ^14^.

A longer aortic cross-clamp time is associated with an increase in the number of patients with POAF. Mathew *et al* reported a 6% increase in incidence of POAF with every fifteen-minute increase in cross-clamp time ^14^. Surgical practices such as bicaval venous cannulation and pulmonary vein venting have been found to significantly increase the incidence of POAF. It is believed that these practices invade the already vulnerable atrium, which causes further discordance in electrical conduction ^14^.

***Post-operative.*** Postoperative risk factors for POAF are less described but several risk factors are linked to POAF especially electrolyte imbalance such as hypomagnesemia and hypokalaemia ^22^. It was noted that 69% of patients that eventually developed AF had hypokalaemia while only 24% of those who did not develop AF had hypokalaemia. Besides that, magnesium supplementation pre-operatively or early post-operative period in hypomagnesemia patients has shown to be preventative of AF. This effect is not apparent in those who have normal magnesium levels post-operatively ^22– 25^.

It is noted that patients with POAF also require higher amount of post-operative usage of inotropic agents ^26^, though it is unclear whether post-operative usage of inotropic agents has a causative mechanism or it is simply used in patients who are relatively unstable and hence are already at a higher risk of developing POAF.

A recent retrospective study also found a strong positive correlation can be seen between the time of POAF onset with the time of maximum blood sugar concentration ^27^.

So far no data have been obtained on the incidence, predictors, and outcome of POAF in our population in Malaysia. This present retrospective study would form the baseline knowledge about this condition, and to compare it with Western figures, and would then form a basis for a possible prospective intervention in trying to reduce the occurrence of POAF.

## Methods

### Study design

This is a single-centre, cross-sectional study of patients who underwent CABG at the National Heart Institute (IJN), Kuala Lumpur, Malaysia. Patient’s medical records were reviewed retrospectively to identify pre-operative factors that predict the development of atrial fibrillation in the post-operative period as well as their outcomes.

### Ethical statement

Ethical approval was obtained through the National Heart Institute Ethics Committee (IJNEC/16/15). No amendments were made throughout the duration of the study. This study was approved by the Monash University Human Research Ethics Committee (CF16/1984 – 2016001004). No patient consent was required by the ethics committees for this study, since there was no patient contact.

### Sample size calculation

The sample size was calculated for this study using the Raosoft® sample size calculator ^28^. The figure of 30% was expected for the incidence of POAF, consistent with previous studies ^9, 10, 12, 13^. With the total number of CABG done in Malaysia being unknown, an overestimated number of 20,000 cases a year was used for the purpose of this calculation. By using the formula for sample size calculation based on the incidence of POAF of 30%, the estimated sample size (n) = Z ^2^P(1-P)/d ^2^, where Z= Z statistic for a level of confidence, P=expected prevalence or proportion, and d=precision ^29^. Therefore, n = [(1.96)2*0.3*0.7]/(0.05) ^2^ = 323. Taking into account a possible 20% attrition rate, the minimum sample size required to determine the incidence of POAF = 323 + (0.2*323) = 388. We managed to obtain data of 637 patients, which further reduced the margin of error of our study to 3.5% with a 95% confidence interval.

### Inclusion and exclusion criteria

Every patient who underwent CABG in IJN over the year of 2015 from January until June was included in this study. Patients who underwent CABG with an additional cardiac surgery in the same sitting were included in the study as well. Conversely, patients who underwent other cardiac surgery without CABG were not included in this study.

The exclusion criteria for this study is patients who had documented AF prior to CABG. Patients who were in sinus rhythm prior to CABG but had previous documented AF, including paroxysmal or intermittent episodes, were excluded from this study as well. This is to ensure that in any case a patient developed POAF, that episode of AF would be his/her first episode or what is known as recent-onset AF. Besides that, patient medical records would need to be complete, with more than half of the variables available for collection. A pre-formed data collection sheet was used to collect all variables ( Supplementary File 1). A total of 680 patients fit the inclusion criteria. 43 patients were rejected in accordance to the exclusion criteria. The remaining 637 patients were included for analysis.

### Statistical analysis

Statistical analysis was carried out using IBM SPSS 23.0.

The prevalence of categorical variables was determined using frequency tables, while continuous variables were described in terms of mean and standard deviation. With these calculations, the general characteristics of the sample was established.

Normality of continuous variables were tested using the degree of skewness and kurtosis. The patients were then grouped according to the onset of POAF and comparison of characteristics between groups were done. Categorical variables were compared using cross tabulations and Chi-square test or Fisher’s exact test was employed to assess significance, whichever more suitable. Student’s t-test was used to compare differences in mean of normal continuous variables between two groups. A *p*-value of less than 0.05 was considered statistically significant.

The secondary outcome of the study in terms of duration of ventilation, hospital stay, ICU stay and high dependency unit (HDU) stay did not follow the normal distribution, hence they were expressed with median value and interquartile range. We decided to establish a cut-off value to delineate between normal and prolonged duration. The cut-off value was determined with reference to hospital protocol.

We cross-tabulated the onset of POAF with the newly categorised groups and utilised Chi-square test or Fisher’s Exact test to assess significance. Additionally, the same analysis was used to compare the prevalence of post-operative complications between patient who developed POAF and patients who did not.

In the last part of the analysis, the group of patients who developed POAF were selected and split into two groups according to the duration of POAF and number of episodes they experienced. The groups were then cross-tabulated with the post-operative complications as well as prolonged ventilation and stay. Chi-square test or Fisher’s exact test were employed to assess significance.

In order to identify potential predictors to be included in the multivariate logistic regression modelling, univariate analysis was performed. The relationship between each pre-operative variable and the onset of POAF was explored. Variables with *p* value of less than 0.25 were included in the multivariate logistic regression. Besides that, variables that were shown to be significant predictors in previous studies were included in the multivariate logistic regression model as well. Variables included in the multivariate logistic regression model were assessed for collinearity.

## Results

### Patients’ characteristics ( Table 1)

The total number of patients included in this study was 637 patients. The patients included in the study sample are pre-dominantly elderly males, representing 82.8% of the cohort with a mean age of 60.66 ± 8.2 years old. The distribution of the patient’s age in our study follows a normal distribution with a minimum age of 30 and a maximum of 91. The majority of the patients (58.2%) were Malays. The Chinese population, standing at 17.6% of our study sample is slightly under represented, while the Indian population is slightly over represented at 22.1% of our sample.

There is a statistically significant difference between the mean age of the groups of patients without POAF and those with POAF (60.00 years vs 62.31 years, *p*=0.002). Besides that, the difference between the non-POAF and POAF group also achieve statistical significance in between the major populations in Malaysia ( *p*=0.001). The Malay population has the highest incidence of POAF at 33.2%, followed by the Chinese population at 27.7%, while the Indian population has the lowest incidence of POAF at 16.3%.

**Table 1.** Characteristics of the sample population in our study and their association with post-operative atrial fibrillation (POAF).

Demographic	Total *, n (%)	Non-POAF group, n (%)	POAF group, n (%)	*p* value
Age (years)	60.66 ± 8.341	60.00 ± 8.316	62.31 ± 8.196	0.002
Male gender	521 (81.8)	376 (82.8)	145 (79.2)	0.289
Malaysian	626 (98.3)	446 (98.2)	180 (98.4)	0.607
Population				0.001
Malay	371 (58.2)	248 (54.6)	123 (67.2)	
Chinese	112 (17.6)	81 (17.8)	31 (16.9)	
Indian	141 (22.1)	118 (26.0)	23 (12.6)	
Others	13 (2.0)	7 (1.5)	6 (3.3)	

*Total n varies slightly for each item due to a small amount of missing data in each.

### Characteristics of POAF ( Table 2)

In total, 183 patients in our study developed AF in the post-operative period, representing 28.7% of the sample. The median time of development of POAF is 45 hours after surgery. The majority of POAF developed within 3-days after surgery with the 2 ^nd^ day being the most common. Only 15% of the POAF in our study developed after more than 3-days post-operation. 95.6% of AF lasts less than 48 hours, while slightly more than one third of the patients had more than one episode of AF in their stay. However, only one patient was discharged with AF in our study, the rest were discharged with sinus rhythm. Of the 91.3% of patients who were treated for AF, amiodarone was the most common intervention, used in 82.5% of the patients. Other interventions used to treat AF were digoxin, beta-blocker and electrical cardioversion.

**Table 2.** Characteristics of post-operative atrial fibrillation (POAF).

Characteristics of AF	n * (%)
Time from surgery to POAF (minutes)	45 ±33
Duration (hours)	
<48	175 (95.6)
>48	8 (4.4)
Number of episodes	
Single	116 (63.4)
Multiple	67 (36.6)
Atrial fibrillation on discharge	1 (0.2)

*Total n varies slightly for each item due to a small amount of missing data in each.

### Pre-operative characteristics ( Table 3)

Considering that Malaysia has one of the highest prevalence of obesity in the world, the median BMI of 26.29 kg/m ^2^ was not unexpected. We noted that 63.7% of the study sample was categorised as overweight and 17.3% as obese, according to the Asian guidelines ^30^. There was no significant difference between the two groups.

**Table 3.** Association between post-operative atrial fibrillation (POAF) and pre-operative characteristics that were recorded.

Pre-operative characteristic	Total *, n (%)	Non-POAF group, n (%)	POAF group, n (%)	*p*-value
Body Mass Index (kg/m ^2^)	26.29 ± 5.09	26.14 ±5.18	26.53 ± 5.20	0.362
< 18.5	4 (0.6)	4 (0.9)	0 (0.0)	
18.5 – 22.9	114 (18.4)	83 (18.8)	31 (17.3)	
23 – 29.9	395 (63.7)	284 (64.4)	111 (62.0)	
>30	107 (17.3)	70 (15.9)	37 (20.7)	
New York Heart Functional Class				0.386
NYHA I	350 (56.7)	243 (55.1)	107 (60.8)	
NYHA II	233 (37.8)	174 (39.5)	59 (33.5)	
NYHA III	34 (5.5)	24 (5.4)	10 (5.7)	
NYHA IV	0 (0.0)	0 (0.0)	0 (0.0)	
Left ventricular ejection fraction	50.5 ± 18	51.0 ± 17	49.0 ± 19	0.035
Left atrial enlargement	180 (28.5)	115 (25.6)	65 (35.7)	0.010
Right atrial enlargement	5 (0.8)	3 (0.7)	2 (1.1)	0.537

*Total n varies slightly for each item due to a small amount of missing data in each

In terms of functional status, the patients in our study have a relatively normal functional status with 350 (56.7%) of the patients classified as New York Heart Functional Class (NYHA) I and 233 (37.8%) classified as NYHA II. By contrast, 34 (5.5%) patients were classified as NYHA III and none classified as NYHA IV. On top of that, the median left ventricular ejection fraction of our patients is 50.5%. There is a statistically significant difference in terms of ejection fraction between groups (51.0% in the non-POAF group vs. 49.0% in the POAF group, *p*=0.035).

There were 180 (28.5%) patients in total who has left atrial enlargement before operation. In total, 115 (25.6%) of them belong to the non-POAF group, while 65 (35.7%) were from the POAF group. The difference between the groups are statistically significant ( *p*=0.010).

### Medical history ( Table 4)

Among the co-morbid conditions that we investigated in our study, hypertension, diabetes mellitus and hypercholesterolemia are the three commonest diagnosed pre-operative co-morbidities in the patients in our study. We noticed 75.4% of our patients had previously been diagnosed with hypertension, 54.8% had diabetes mellitus and 69.5% had hypercholesterolemia. In total, 276 patients (43.3%) were previous or current smokers. Amongst all the investigated pre-operative medical conditions, chronic kidney disease (CKD) and end-stage renal failure with dialysis were the two conditions that observed a statistically significant difference between two groups. A total of 26 patients with previously diagnosed CKD also developed POAF (14.2%), compared to the 28 patients who did not develop POAF (6.2%). Similarly, 11 patients who underwent dialysis due to end stage renal failure before operation developed POAF, compared to the 6 who did not develop POAF.

**Table 4.** Association between post-operative atrial fibrillation (POAF) and underlying medical conditions on admission.

Medication	Total *, n (%)	Non-POAF group, n (%)	POAF group, n (%)	*p* value
Chronic obstructive pulmonary disease	11 (1.7)	10 (2.2)	1 (0.5)	0.192
Asthma	25 (3.9)	20 (4.4)	5 (2.7)	0.377
Hypertension	480 (75.4)	342 (75.3)	138 (75.4)	0.983
Diabetes Mellitus	349 (54.8)	247 (54.4)	102 (55.7)	0.760
Hypercholesterolemia	443 (69.5)	313 (68.9)	130 (71.0)	0.603
Chronic kidney disease	54 (8.5)	28 (6.2)	26 (14.2)	0.001
End stage renal failure with dialysis	17 (2.7)	6 (1.3)	11 (6.0)	0.001
Current or ex-smoker	276 (43.3)	203 (44.7)	73 (39.9)	0.266
Alcohol	47 (7.4)	33 (7.3)	14 (7.7)	0.868

*Total n varies slightly for each item due to a small amount of missing data in each

### Pre-operative medications ( Table 5)

The medications list tabulated below reflects the treatments given to our patient’s underlying medical condition. Anti-hypertensive, anti-lipid, anti-platelets and anti-angina agents were present in more than half of our patients’ medication list. The abundant prescription of these medications seen in our patients is not surprising as these are the staple medications used in cardiovascular diseases. Amongst the medications, beta-blocker is the only medication that has a statistical significant difference between the two groups. In total, 292 patients (64.3%) of the patients in the non-POAF group were prescribed beta blockers, while 134 (73.2%) of the patients in the POAF group were prescribed beta-blockers ( *p*=0.031).

**Table 5.** List of patient medications on admission, classified into groups. Association between post-operative atrial fibrillation (POAF) and individual medication is presented.

Pre-operative medication	Total *, n (%)	Non-POAF group, n (%)	POAF group, n (%)	*p-*value
Anti-hypertensive				
ACE inhibitor	306 (48.0)	219 (48.2)	87 (47.5)	0.873
Angiotensin Receptor Blocker	98 (15.4)	68 (15.0)	30 (16.4)	0.654
Calcium Channel Blocker	159 (25.0)	107 (23.6)	52 (28.4)	0.201
Beta-Blocker	426 (66.9)	292 (64.3)	134 (73.2)	0.031
Other Anti-hypertensives	45 (7.1)	36 (7.9)	9 (4.9)	0.180
Anti-platelet				
Aspirin	460 (72.2)	333 (73.3)	127 (69.4)	0.314
Clopidogrel	292 (45.8)	210 (46.3)	82 (44.8)	0.740
Other anti-platelets	107 (16.8)	74 (16.3)	33 (18.0)	0.596
Anti-lipid				
HMG CoA Inhibitor (statins)	570 (89.5)	409 (90.1)	161 (88.0)	0.432
Fibrates	22 (3.5)	16 (3.5)	6 (3.3)	0.878
Ezetimibe	23 (3.6)	15 (3.3)	8 (4.4)	0.513
Anti-diabetic				
Biguanide	236 (37.0)	178 (39.2)	58 (31.7)	0.076
Sulphonylurea	135 (21.2)	97 (21.4)	38 (20.8)	0.867
Alpha Glucosidase Inhibitor	4 (0.6)	3 (0.7)	1 (0.5)	0.869
DPP-4 Inhibitor	61 (9.6)	42 (9.3)	19 (10.4)	0.664
Insulin	136 (21.4)	91 (20.0)	45 (24.6)	0.205
Inhaled agents				
Inhaled Beta Agonist	28 (4.4)	24 (5.3)	4 (2.2)	0.084
Inhaled Anti Muscarinic	15 (2.4)	12 (2.6)	3 (1.6)	0.450
Inhaled Steroids	17 (2.7)	14 (3.1)	3 (1.6)	0.306
Anti-angina	428 (67.2)	306 (67.4)	122 (66.7)	0.858
Diuretics	133 (20.9)	86 (18.9)	47 (25.7)	0.058

*Total n varies slightly for each item due to a small amount of missing data in each

### Operative details ( Table 6)

The commonest performed surgery in our patients is CABG alone, performed in 586 patients (92.0%). Compound surgeries are relatively uncommon, only performed in 51 of the cases (8.0%). Amongst the compound surgeries, CABG and valve is the most commonly performed, representing 6.3% of the total surgeries performed. Mitral valve surgery is the most commonly performed valve surgery, representing 61.4% of the valve surgery performed with CABG in our patients.

**Table 6.** Association between post-operative atrial fibrillation (POAF) and patient operative details. CABG, coronary bypass grafting.

Operative details	Total *, n (%)	Non-POAF group, n (%)	POAF group, n (%)	*p*-value
Surgery type				0.025
CABG alone	586 (92.0)	424 (93.4)	162 (88.5)	
CABG + valve	40 (6.3)	23 (5.1)	17 (9.3)	
CABG + other	7 (1.1)	6 (1.3)	1 (0.5)	
CABG + valve + other	4 (0.6)	1 (0.2)	3 (1.6)	
Cardiopulmonary bypass				0.351
On pump	608 (95.4)	430 (94.7)	178 (97.3)	
Off pump	25 (3.9)	21 (4.6)	4 (2.2)	
On pump beating heart	4 (0.6)	3 (0.7)	1 (0.5)	
Bypass time	82 ± 52	82 ± 51	83 ± 60	0.570
Cross-clamp time	64 ± 46	64 ± 43	63 ± 53	0.591

*Total n varies slightly for each item due to a small amount of missing data in each

The vast majority of the patients underwent on-pump CABG (608 patients, representing 95.4% of the total). Amongst them, 178 patients were in the POAF group, representing 97.3% of the POAF group; 430 patients were in the non-POAF group, representing 94.7% of the non-POAF group. However, the difference is not statistical significant. A total of 25 patients underwent off-pump CABG (3.9), while 4 patients underwent on pump beating heart CABG (0.6%).

The median bypass time is 82 minutes, with a minimum bypass time of 32 minutes and a maximum bypass time of 300 minutes. On the other hand, the median cross-clamp time is 64 minutes, with a minimum cross-clamp time of 22 minutes and a maximum cross-clamp time of 225 minutes. There was no statistically significant difference between the two groups in terms of bypass and cross-clamps times.

### Identifying predictors ( Table 7)

Univariate analysis was performed on every recorded variable to identify potential predictors to be included in the multivariate model. Age, Malay population, Indian population, history of CKD, left ventricular ejection fraction, beta-blocker, biguanide, left atrial enlargement and CABG plus valve surgery were identified as significant predictors in the univariate analysis ( *p*<0.05).

Age, Indian population, history of CKD, left ventricular ejection fraction and beta blocker were identified as independent predictors in our study after multi-variable adjustments. With every additional year a patient advances, there is a 4.5% additional odds of developing POAF (AOR=1.045, 95% CI, 1.019 – 1.072, *p*=0.001). The Indian population is less likely to develop POAF compared to the other populations, with a 56% reduction in odds (AOR=0.440, 95% CI, 0.253 – 0.765, *p*=0.004). The Malay population had a significant association with POAF in the univariate analysis, but the significance was lost after adjustment.

**Table 7.** Relationship between post-operative atrial fibrillation (POAF) and selected variables before and after adjustments. CABG, coronary bypass grafting.

	Crude OR (95% CI)	*p*-value	Adjusted OR (95%)	*p*-value
Age	1.035 (1.013 – 1.058)	0.002	1.045 (1.019 – 1.072)	0.001
Malay population	1.703 (1.188 – 2.440)	0.004		
Indian population	0.409 (0.252 – 0.665)	>0.001	0.440 (0.253 – 0.765)	0.004
Chronic obstructive pulmonary disease	0.244 (0.031 – 1.192)	0.180		
Hypertension	1.004 (0.674 – 1.496)	0.983		
Diabetes Mellitus	1.055 (0.747 – 1.490)	0.760		
Hypercholesterolemia	1.105 (0.758 – 1.610)	0.603		
Chronic kidney disease	2.619 (1.483 – 4.625)	0.001	2.124 (1.057 – 4.268)	0.034
Smoker	0.821 (0.579 – 1.163)	0.267		
Ejection fraction	0.984 (0.969 – 0.999)	0.033	0.977 (0.958 – 0.996)	0.016
Body Mass Index	1.026 (0.982 – 1.072)	0.246		
Calcium channel blocker	1.281 (0.874 – 1.897)	0.201		
Beta-blocker	1.517 (1.038 – 2.217)	0.031	1.611 (1.049 – 2.467)	0.029
HMG CoA inhibitor	0.805 (0.469 – 1.384)	0.433		
Biguanide	0.076 (0.500 – 1.035)	0.076		
Insulin	1.301 (0.865 – 1.955)	0.206		
Diuretics	1.479 (0.985 – 2.220)	0.059		
Inhaled beta agonist	0.400 (0.137 – 1.170)	0.094		
Left atrial enlargement	1.618 (1.118 – 2.343)	0.011		
Off-pump CABG	0.460 (0.156 – 1.360)	0.160		
Bypass time	1.003 (0.999 – 1.007)	0.125		
Cross-clamp time	1.003 (0.998 – 1.008)	0.197		
CABG+valve surgery	1.994 (1.015 – 3.917)	0.045		
CABG+valve+other surgery	7.852 (0.811 – 76.032)	0.075		

History of CKD is another significant predictor of POAF as patients with CKD have more than double the odds of developing POAF (AOR = 2.124, 95% CI, 1.057 – 4.268, *p*=0.034). Left ventricular ejection fraction also has a significant association with POAF. With every 10% increment in the ejection fraction value, there is a 23% reduction of odds of developing POAF (AOR=0.977, 95% CI, 0.958 – 0.996, *p*=0.016). Lastly, pre-operative beta-blocker treatment has a positive association with POAF, as well with a 61% increase in odds over those who were not under beta-blocker treatment (AOR = 1.611, 95% CI, 1.049 – 2.467, *p*=0.029).

### Post-operative outcomes ( Table 8)

The mortality rate of our study stands at 2.7%. The mortality rate of the non-POAF group is 1.8% compared to 4.9% in the POAF group. There is a statistically significant difference between the mortality rates of both groups.

**Table 8.** Association between post-operative atrial fibrillation (POAF) and post-operative outcomes.

Post-operative outcomes	Total *, n (%)	Non-POAF group, n (%)	POAF group, n (%)	*p*-value
Reoperation	59 (9.3)	29 (6.4)	30 (16.4)	<0.001
Stroke	4 (0.6)	1 (0.2)	3 (1.6)	0.074
Renal failure requiring dialysis	21 (3.3)	9 (2.0)	12 (6.6)	0.003
Pulmonary complications	51 (8.0)	29 (6.4)	22 (12.0)	0.018
Gastro-intestinal complications	18 (2.8)	11 (2.4)	7 (3.8)	0.334
Surgical infections	49 (7.7)	30 (6.6)	19 (10.4)	0.106
Pericardial effusion	40 (6.3)	25 (5.5)	15 (8.2)	0.205
Fever	81 (12.7)	48 (10.6)	33 (18.1)	0.010
Death	17 (2.7)	8 (1.8)	9 (4.9)	0.025

*Total n varies slightly for each item due to a small amount of missing data in each

Four patients in total developed stroke in the post-operative period. Three patients are from the POAF group and one from the non-POAF group, yielding incidence of stroke of 1.6% and 0.2%, respectively. However, the difference of stroke incidence between the two groups did not achieve statistical significance.

On top of that, 30 patients (16.4%) in the POAF group were re-operated while 29 patients (6.4%) in the non-POAF group were re-operated, a statistically significant difference was observed. Other post-operative complications that achieved statistical significant difference between two groups are renal failure requiring dialysis (2.0% in non-POAF group vs. 6.6% in the POAF group, *p*=0.003), pulmonary complications (6.4% in non-POAF group vs. 12.0 in the POAF group, *p*=0.018) and post-operative fever (1.8% in non-POAF group vs. 4.9% in POAF group, *p*=0.025).

### Post-operative stay ( Table 9)

The differences between the duration of ICU stay, HDU stay and hospital stay between the two groups were statistically significant. The median duration of ICU stay including readmission was 1648 minutes in the non-POAF group, compared to 3542 minutes in the POAF group, *p*<0.001. A total of 100 patients had prolonged ICU stay in the POAF group, accounting for more than half of the patients with POAF (54.9%). On the flipside, 136 patients in the non-POAF group had a prolonged ICU stay, accounting to 30.2% of those who did not develop POAF, *p*<0.001. 

**Table 9.** Association between post-operative atrial fibrillation (POAF) and duration of stay, as well as duration of ventilation. ICU, intensive care unit; HDU, high dependency unit.

Durations	Total *, n (%)	Non-POAF group, n (%)	POAF group, n (%)	*p-*value
Duration in ICU in minutes	1766 ± 2798	1648 ± 1615	3542 ± 4546	<0.001
Prolonged ICU stay	236 (37.3)	136 (30.2)	100 (54.9)	<0.001
Duration in HDU in minutes	1680 ± 1515	1590 ± 1450	2430 ± 2575	0.003
Prolonged HDU stay	74 (26.9)	36 (21.4)	38 (35.5)	0.010
Duration of ventilation in minutes	1200 ± 365	1155 ± 363	1277.5 ± 584	<0.001
Prolonged ventilation	139 (22.0)	77 (17.1)	62 (34.1)	<0.001
Duration of hospital stay in days	7.3 ± 2.9	7.2 ± 2.1	9.0 ± 5.9	<0.001
Prolonged hospital stay	63 (9.9)	26 (5.7)	37 (20.2)	<0.001
Readmission into ICU	17 (2.7)	8 (1.8)	9 (4.9)	0.029
Readmission into HDU	4 (0.6)	2 (0.4)	2 (1.1)	0.327
Hospital readmission	15 (2.4)	13 (2.9)	2 (1.1)	0.147
Reintubation	11 (1.7)	2 (0.4)	9 (4.9)	<0.001

*Total n varies slightly for each item due to a small amount of missing data in each

The median duration of HDU stay including readmission in the non-POAF group was 1590 minutes, compared to 2430 minutes in the POAF group, *p*=0.003. In total, 38 patients (35.5%) experienced a prolonged HDU stay in the POAF group, compared to 36 (21.4%) in the non-POAF group, *p*=0.010.

The median duration of hospital stay was 7.2 days in the non-POAF group, while patients in the POAF group had a median duration of stay in the hospital of 9.0 days ( *p*<0.001). A total of 37 patients (5.7%) in the POAF group had prolonged hospital stay, compared to 26 (20.2%) from the non-POAF group ( *p*<0.001).

The duration of ventilation including reintubation was significantly different statistically in both groups as well. The median duration of ventilation in the non-POAF patients was 1155 minutes, compared to the 1277.5 experienced by the patients with POAF. In total, 62 (34.1%) patients had prolonged ventilation in the POAF group compared to 77 (17.1%) in the non-POAF group.

There is a significant difference in the incidence of readmission into ICU between the two groups. In total, 9 patients were readmitted into the ICU (4.9%) in the POAF group, while 8 patients were readmitted in the non-POAF group (1.8%), *p*=0.029. But the readmission rates into the HDU and hospital were not statistically significant between the two groups. However, reintubation rates between the two groups were significantly different statistically with a 4.9% reintubation rate in the POAF group and 0.4% reintubation rate in the non-POAF group, *p*<0.001.

Raw data for the study ‘A retrospective study on atrial fibrillation after coronary artery bypass grafting surgery at The National Heart Institute, Kuala Lumpur’ both in SAV and Excel formats Click here for additional data file. Copyright: © 2018 Farouk Musa A et al. 2018 Data associated with the article are available under the terms of the Creative Commons Zero "No rights reserved" data waiver (CC0 1.0 Public domain dedication).

## Discussion

AF is one of the most common adverse events after cardiac surgery occurring in slightly more than a quarter of patients in our study. Reported incidences range from 10–65%, depending on patient profile, type of surgery, method of arrhythmia surveillance, and definition of arrhythmia ^31– 33^.

While the precise pathophysiology of POAF is unknown, most of the evidence suggests that it is multifactorial. A common underlying factor associated with POAF induced by mechanical, metabolic or pharmacologic stimuli is the redox changes in atrial tissue associated with tachyarrhythmia ^34^.

POAF adversely affects mortality and morbidity, and consequently leads to a longer hospital stay and higher costs related to the cost of care ^26, 35^. There are certain pre-operative factors that predict the development of POAF. In our study, we found that advancing age, history of CKD, non-Indian populations, low left ventricular ejection fraction and pre-operative beta-blocker treatment are independently associated with the development of POAF.

The strength of our study lies on the large sample size with a good racial distribution among the three major populations in Malaysia, allowing good representation of the multiracial background. This being the first study of its kind in Malaysia provides a good background of the incidence of POAF and its outcomes for future studies in this country.

### Incidence of POAF in Malaysia

The incidence of POAF at the National Heart Institute (IJN), Malaysia, stands at 28.7% in the present study, which is within the range of most large series that report an incidence of around 30% ^26^. Since this is, to our knowledge, the first study in Malaysia to investigate POAF, we do not have any previous studies to compare our figures to.

Singapore is a country that lies in close proximity to Malaysia, shares the same multiracial population background as Malaysia and serves as a good point for comparison. In a recent prospective study conducted in heart institutes in Singapore, the recorded incidence of POAF was 17.3%, much lower than the incidence of 28.7% observed in our study. One possible explanation to this disparity between the incidences could be due to the relatively stringent criteria employed by the investigators in that study. Only AF episodes lasting more than 1 hour were considered as POAF in that study, whereas we included every episode of documented AF regardless of duration ^36^.

Compared to other studies conducted mainly in the Western population, our incidence of POAF is similar to those conducted in the United Kingdom, Europe and the United States ^35^. The findings from our study supports the notion that POAF is a commonly occurring arrhythmia in the post-operative period.

### Characteristics of POAF

The characteristics of POAF that were described in previous studies were transient episodes of AF, usually occurring between two to four days after operation with a high rate of recurrence ^37, 38^. The characteristics of POAF episodes observed in our study is similar to the description from previous studies.

In our study, the median time of development of POAF was 45 hours after operation with the majority of the first POAF episodes happen within three days after operation. The recurrence rate of POAF was 36.6%, comparable to the 40% recurrence rate seen in previous studies ^37, 38^. Knowing the characteristics of POAF will allow treating healthcare professionals to anticipate POAF better and be more vigilant in terms of monitoring and management.

We decided to select 48-hours as the POAF duration cut-off to categorize patients in favor of the more commonly used 24-hour cut-off period in an attempt to identify patients who required prophylactic anti-coagulation. In total, 8 of our patients had AF duration of more than 48-hours and were recommended to be started on prophylactic anti-coagulation for thromboembolic prevention in accordance to the management guideline ^39, 40^.

In addition, 2 patients passed away in the post-operative period, 3 were not started on any anti-coagulation therapy and the other 3 were started on warfarin, but this was most likely due to their respective valve replacements. The conservative approach of the treating team in IJN in terms of prophylactic anti-coagulation is most likely due to the relatively low incidence of stroke weighed against the high incidence of post-operative bleed in this hospital.

### Pre-operative predictors

Univariate analysis was performed to every recorded pre-operative and operative variable in an attempt to identify potential predictors to be included in the final multivariate model. Variables that had significant association with POAF before adjustments were age, Malay population, Indian population, history of CKD, left ventricular ejection fraction, beta-blocker, biguanide, left atrial enlargement and CABG plus valve surgery ( *p*<0.05) Other variables that were identified as potential predictors ( *p*<0.25) include history of COPD, BMI, calcium channel blocker, insulin, diuretics, inhaled beta agonist, off-pump CABG surgery, bypass time, cross-clamp time and CABG plus valve plus other surgery.

All of the potential predictors that were mentioned above were included in the final multivariate logistic regression analysis to identify independent predictors. Hypertension, diabetes mellitus, hypercholesterolemia, smoking status and HMG CoA were included in the multivariate logistic model, as they were shown in previous studies ^35– 37^ to be significant independent predictors of POAF.

After adjustment, age, Indian population, history of CKD, left ventricular ejection fraction and beta-blocker remained as variables with independent association with POAF.

***Age***. Advancing age has been one of the most described pre-operative risk factor for POAF with strong associations established in previous studies. Mathew *et al* estimates that in patients over the age of 70, every 10-year increment in age will yield a 75% increase in the odds of developing POAF ^35^. The associations were not as strong in our model, with a 45% increase in odds of developing POAF for every decade of increment in patient’s age. However, we would like to note that our model is not age restricted.

It is not surprising that the association between advancing age and AF is not restricted to the post-operative period. A strong association between advancing age and AF is seen in the general population as well with a higher incidence of 2–4% seen in the elderly population compared to the 0.4% seen in the younger population ^40^. The strong association is due to the age-related changes in cardiac anatomy seen in the elderly, making them more prone to develop AF ^41^. Along with a higher incidence of cardiac related co-morbid conditions acting as stressors to the heart, cardiac surgeries act as triggers to the eventual development of AF in the elder population.

***Indian population.*** With the majority of the POAF-related studies conducted in Western countries, the association between the multiracial background in Asian countries like Malaysia is still relatively unknown. Our study found a significantly lower-odds of developing POAF in the Indian population compared to their counterparts of other racial origins. The study conducted in Singapore found a similar trend, reporting a higher-odds of developing POAF in patients from Chinese and Malay populations compared to those of Indian ethnicity. To our knowledge this is the only other study that investigated this association ^36^.

The relative resistance of developing POAF observed in the Indian population in our study could be traced back to their genetic lineage from India. According to a multinational study, the reported incidence of POAF in India was 15.7%, almost half of the incidence of POAF in Malaysia ^35^. On top of that, the prevalence of AF in the general population in India is relatively low as well, with an overall incidence of 0.39% as reported by the PINNACLE registry ^42^. More studies, both clinical and scientific, needs to be done to identify the reason behind this resistance of AF seen in the Indian population. Exploring this subject might bring about new theories on the still relatively unknown pathogenesis of POAF.

***Chronic kidney disease (CKD)***. Among other pre-operative co-morbid conditions investigated in our study, CKD is the only one with significant association after adjustments. Patients with a history of CKD are more than two times more likely to develop POAF compared to those with normal kidney function (AOR = 2.124, 95% CI, 1.057 – 4.268, *p*=0.034). This association is consistent with those found in previous studies ^26, 43^.

The strong association found between CKD and AF in this study is most probably due to the pro-inflammatory state experienced by CKD patients. In fact, this association is not limited to chronic form of kidney disease. A recent study investigating the relationship between POAF and post-operative acute kidney failure found a significant association as well, suggesting a possible linkage between the pathogenesis of AF between the acute and chronic form of kidney disease ^43^.

***Left ventricular ejection fraction***. Left ventricular ejection fraction is an estimate of the functioning status of the heart. Since congestive heart failure was described in previous study to be associated with an increased-odds of developing POAF, it does not come as a surprise that a high ejection fraction, indicating a high level of functioning of the heart, reduces the odds of developing POAF.

***Beta-blocker***. Lastly, the surprising positive association between pre-operative beta-blocker treatment and POAF was found in our study. Patients who were prescribed beta-blockers were 61% more likely to develop POAF compared to those who were not prescribed beta-blockers. This result is in contradiction to other studies and meta-analyses ^44^ that concluded that beta-blockers are effective in reducing POAF if given in the prophylactically in the pre-operative period. In fact, guidelines have recommended the prescription of beta-blockers to low-risk individuals to prevent POAF.

One of the possible explanations of this phenomenon could be due to the rebound effect when patients were taken off beta-blockers during the post-operative period. Usually the recommencement of beta-blockers in the post-operative period is dependent on the preference of the treating team and the stability of the patient. In fact, the cessation of beta-blocker was one of the risk factors of developing POAF ^35^. Unfortunately, one of the limitations of our study was the lack of detection of the recommencement of beta-blockers in the post-operative period, rendering our study underpowered to confirm this association.

***Other variables***. Certain variables, such as left atrial enlargement, longer bypass time and longer cross-clamp times, were associated with an increased-odds of developing POAF in previous studies. However, the association between these variables and POAF was not found to be significant in our study after adjustments.

Pre-operative co-morbid conditions, such as hypertension, diabetes, hypercholesterolemia and COPD, were also shown in previous studies to be independent predictors of POAF ^45– 47^; however this association could not be established in our study.

Although the incidence of POAF in patients who underwent compound surgeries were higher compared to those who underwent CABG alone, the association was not significant after adjustment. However, we noted that the proportion of patients who underwent compound surgery was much less compared to those who underwent CABG alone. On top of that, it is likely that patients who are undergoing compound surgery, especially mitral valve related, to have underlying AF and were subsequently excluded from our study.

### Outcomes

***Mortality***. The mortality rate observed in our study is 2.7%, slightly lower than the mortality rates seen in other studies. The mortality rate in the POAF group is 4.9%, compared to the 1.8% seen in the non-POAF group, a statistically significant difference. This was about three-fold increase in mortality rate in AF patients compared with patients without AF.

A similar difference in mortality rates were seen in other studies ^7, 48, 49^. POAF does not only affect the short-term mortality but long-term mortality as well. This finding demonstrates the not-so-benign nature of this arrhythmia.

It is thought that the mechanisms by which postoperative AF is associated with mortality are speculative. This might be due to hemodynamic compromise and heart failure directly as a result of the loss of atrial transport function may certainly contribute. In the long term, mechanistic and causal links are more difficult to establish. Possibilities include the development of heart failure with its attendant mortality risk, the occurrence of disabling stroke or other embolic catastrophes, and adverse drug effects, such as pro-arrhythmia with antiarrhythmic drugs or haemorrhage with anticoagulants.

***Morbidity***. We observed that all of the investigated post-operative complications have higher rates in the POAF group compared to the non-POAF group. One of the most devastating complications of morbidity due to AF is the increased incidence of stroke. This is thought to be largely due to circulatory stasis in the left atrium resulting in the formation of an embolus.

There was a six-fold increase in the incidence of stroke, one of the feared sequelae of AF, in the POAF group compared to the non-POAF group (1.6% vs 0.2%). However, the difference did not achieve statistical significance due to the low incidence seen in this study.

Attempts to investigate other AF-related complications, such as post-operative myocardial infarction and new heart failure, were quickly abandoned as we realized that a sufficiently accurate representation cannot be obtained from the medical records.

The investigated outcomes that achieved statistical significance are reoperation, renal failure requiring dialysis, pulmonary complications and post-operative fever. Due to the nature of our study, the interpretation of the outcomes with POAF needs to be perform with caution as a temporal relationship cannot be established. Since the development of AF does not affect the pathogenesis of these outcomes directly, the notion that these complications may trigger the development of AF cannot be ruled out. These complications are generally pro-inflammatory, on top of that patients who underwent reoperation are exposed to more inotropic agents and surgical insults while patients with renal failure are more likely to have electrolyte imbalance and fluid overload, conditions of which may play a role in the pathogenesis of AF.

***Post-operative stay***. From our study, we noticed that patients with POAF experience significantly longer stay in the ICU, HDU and the hospital ( *p*<0.001). Besides that, the duration of ventilation experienced by patients in the POAF group was significantly longer as well. Extended period of ICU and HDU stay for post-op AF patients may be due to further observation, management and nursing needed for stabilization of haemodynamic status, correction of hypoxia and for conversion of AF status to sinus rhythm.

In addition, there were higher rates of readmission into ICU, reintubation, readmission into HDU and hospital readmission in the POAF group, with the former two achieving statistical significance. This is common due to the nature of patients who developed AF after surgery are prone to develop other morbid events that need extended care facility, such as ventilatory support. Consequently, this led to a higher usage of hospital resources by the patients with POAF due to the prolonged stay and ventilation, which translates into increased cost to both the patients and the hospital.

### Limitations

The main limitation to this study lies in its observational nature. As such, temporal relationship was unable to be established on certain variables, especially the post-operative outcomes with regards to post-operative AF. Besides that, the amount of data collected in this study is strictly limited to the amount recorded in the patient medical records. Certain variables that will take our study a step above such as AF related complications, AF treatment related complications, and exact left atrial sizes were simply unavailable or inconsistently documented. We also did not take some possible predictive factors such as the state of autonomic nervous system ^50, 51^ as independent predictors of POAF since this is a retrospective review and we did not monitor such changes in our patients.

Besides that, another limitation to our study would be the direct role the investigator played during the data collection process, giving rise to the possibility of observer bias. However, in order to reduce the influence of bias, part of the list of variables were collected by blinded data collectors, away from the influence of the investigator.

### Future directions

Moving forward, to obtain higher quality data regarding the incidence of POAF with their respective outcome, a large prospective cohort study with long term follow-up would be indicated. Ideally, patients would be put on 24-hour cardiac monitoring for the first week to detect episodes of AF. Vital signs and post-operative complications have to be charted and cross checked with the time of onset of POAF to detect any trends or patterns. Date of cessation and recommencement of medication will be recorded. Patients will also have to be followed-up over years to detect the incidence of late AF. Resource utilization and cost will probably have to be recorded to allow accurate cost effectiveness analysis.

Besides that, randomized controlled trials can also be conducted to investigate potential preventative interventions. Certain preventative interventions have yielded promising results in earlier studies ^52– 57^, more randomized controlled trials may give us enough evidence to adopt new methods in the prevention of POAF. Perhaps prevention of POAF using extracts from natural products like tocotrienol, which is a vitamin-E isomer derived from Palm Oil, that possesses potent anti-oxidative properties sounds promising ^56, 57^.

Furthermore, healthcare professionals related to this field should be made aware of the potential implications of POAF on their patients’ health. Local management guidelines and protocols should be modified to reflect the findings of this study.

## Conclusions

AF is the most common complication after cardiac surgery and is associated not only with increased morbidity and mortality, but also with increased costs and longer hospital stay. A number of preoperative patients’ characteristics and intraoperative practice variables appear to affect the incidence of this arrhythmia.

Pre-operative and operative predictors identified in this study are consistent with the findings of studies conducted in Western countries. The multiracial population in Malaysia produced a unique study question. Previous conclusion of Asians having a lower incidence of POAF compared to Westerners will need to be revisited as our study suggests that the resistance to POAF is only limited to the Indian population.

The measurable outcomes of care, such as the need for re-intubation, ventilatory support, longer ICU, HDU and hospital stay, are all associated with postoperative AF, leading to an increase in resource utilization. Strategies to identify the patients at risk and to modify these risk factors by aggressive prophylactic measures, as well as changes in surgical techniques, should lead to lower incidence of AF and a reduced morbidity and mortality rate for patients undergoing cardiac surgery.

We hope that this study can spark a wave of interest on the subject of POAF as this condition has and will continue to affect the lives of patients. This study has highlighted the need of high quality studies and the need for prevention of this condition that is associated with an increase in morbidity and mortality, from the healthcare providers, especially in Malaysia.

Therefore, we recommend a prospective randomized control trial on the preoperative identification and prophylactic intervention in order to develop an efficient prevention and management strategies in reducing the incidence of POAF.

## Data availability

The data referenced by this article are under copyright with the following copyright statement: Copyright: © 2018 Farouk Musa A et al.

Data associated with the article are available under the terms of the Creative Commons Zero "No rights reserved" data waiver (CC0 1.0 Public domain dedication). 

Dataset 1: Raw data for the study ‘A retrospective study on atrial fibrillation after coronary artery bypass grafting surgery at The National Heart Institute, Kuala Lumpur’ both in SAV and Excel formats. DOI, 10.5256/f1000research.13244.d192982 ^58^.

## Supplementary material

Supplementary File 1: Pre-formulated data collection sheet.
