# Supplementary material for: A retrospective study on atrial fibrillation after coronary artery bypass grafting surgery at The National Heart Institute, Kuala Lumpur
Source: F1000Res. 2018 Aug 1;7:164. Originally published 2018 Feb 8. [Version 2] doi: 10.12688/f1000research.13244.2 (PMC6127737; doi:10.12688/f1000research.13244.2)
Supplement: Supplementary file 2 [file f1000research-7-16902-s0001.tgz › ea3998ac-96c5-4777-af97-3fd62c4f09aa.pdf]

<u>Study Proforma for the project:</u>

A Single Centre Retrospective Review on Atrial Fibrillation After Coronary Artery Bypass Grafting

1. Name
2. RN
3. Sex
4. Race
5. Age
6. Date of Admission: dd/mm/yyyy
7. Date of Surgery: dd/mm/yyyy
8. Date of Discharge: dd/mm/yyyy

9. Pre-morbid conditions: (Yes/No)
   i. HPT
   ii. DM
   iii. Hypercholesterolaemia
   iv. COPD
   v. CHF
   vi. Chronic AF
   vii. Others: Specify
10. Pre-surgery medications (Yes/No. If Yes please specify name of medication)
    i. Anti hypertensive
    ii. Anti platelets (Aspirin/Cardiprin/Clopidogrel)
    iii. Anti lipids
    iv. Oral hypoglycaemics
    v. Insulin
    vi. NSAIDs
    vii. Others: Specify
11. NYHA Pre-Op: I, II, III, IV
12. ECHO findings: (Normal/Abnormal. If Abnormal please state the value)
    i. Left atrium size
    ii. Right atrium size
    iii. Ejection fraction

13. Type of CABG:
    i. On-Pump
    ii. Off-Pump
14. CABG Procedures
    i. Isolated
    ii. With Valve Procedure
        a. Mechanical valve
        b. Prosthetic valve

15. Cross clamp time: minutes
16. Bypass time: minutes

17. Ventilation time

18. CICU Ventilation
    i. Initial hours ventilation
    ii. Reintubation
    iii. Total hours ventilated
19. Total CICU stay
    i. Initial hours CICU stay
    ii. Readmission
    iii. Total hours CICU stay

20. Total HDU stay
    i. Initial hours HDU stay
    ii. Readmission
    iii. Total hours in HDU

21. Onset of AF (Yes/No. If Yes please specify how many hours post op)
    i. On-pump CABG: Hours post-op
    ii. Off-pump CABG: hours post-op

22. Treatment for AF
    i. Amiodarone
    ii. Digoxin
    iii. Beta-blocker
    iv. Electrical cardioversion
    v. Others: Specify

23. Total duration of hospital stay: days

24. Morbidities associated with AF post CABG (Yes/No)
    i. Stroke
    ii. MI
    iii. Infection
    iv. Respiratory problems
    v. Renal failure
    vi. Gastrointestinal problems
    vii. Endocrine problems
    viii. Others: please specify

25. Death

26. Discharged
    i. Rhythm on Discharged:
        a. Sinus rhythm
        b. AF
        c. Others: Specify
    ii. Antiarrhythmic agents:
        a. Amiodarone
        b. Beta blockers

c. Others: Specify

iii. Medications on Discharged:

a. Aspirin

b. Warfarin

c. Anti platelets

d. Statins

e. Diuretics

f. Digoxin

g. Others: Specify

27. Follow-Up

i. ECG on Follow up

a. Sinus rhythm

b. AF

c. Others: Specify

iii. Anti-arrhythmic drugs

a. Amiodarone

b. Beta-blocker

c. Others: Specify

iv. Anti-coagulation:

a. Warfarin

b. Others: Specify

28. Readmission

i. Date of readmission: dd/mm/yyyy

ii. Reason for readmission

29. Re-do surgery

i. Date of re-do surgery: dd/mm.yyyy
