# Supplementary material for: A retrospective study on atrial fibrillation after coronary artery bypass grafting surgery at The National Heart Institute, Kuala Lumpur
Source: F1000Res. 2018 Aug 1;7:164. Originally published 2018 Feb 8. [Version 2] doi: 10.12688/f1000research.13244.2 (PMC6127737; doi:10.12688/f1000research.13244.2)
Supplement: Peer review history

# Peer review: Referee response for version 2

**DOI:** 10.5256/f1000research.16902.r36675

**Published:** 2018-09-05

**Authors:** Vlasta Bari

**Affiliations:** 1 Department of Cardiothoracic and Vascular Anesthesia and Intensive Care, IRCCS Policlinico San Donato, Milan, Italy

I have read this submission. I believe that I have an appropriate level of expertise to confirm that it is of an acceptable scientific standard.

# Peer review: Referee response for version 1

**DOI:** 10.5256/f1000research.14368.r33419

**Published:** 2018-05-30

**Authors:** Vlasta Bari

**Affiliations:** 1 Department of Cardiothoracic and Vascular Anesthesia and Intensive Care, IRCCS Policlinico San Donato, Milan, Italy

Authors propose a retrospective study about the incidence of post operative atrial fibrillation (POAF) in patients undergoing coronary artery bypass surgery in Malaysia.

 To do so, 637 medical records have been considered, including several confounding factors that could be determinant of the outcome.

 Results show that the incidence of POAF in Malaysis is similar to the European one.

 The paper is well written and factors are correctly described.

 Methods and statistics are appropriate.

 I only have some concerns:

- The paper is quite long and it could be considered to try to revise some parts. I would like to suggest the authors try to revise some paragraphs, discarding the less necessary and focusing on the more important information, in order to improve readability
- To strengthen the association with predictive factors of POAF it would be interesting to see also ROC curve showing the value of the area under the curve. That could result from the multivariate logistic regression analysis determined by including all factors resulting predictive for POAF.
- Some possible predictive factors, as the state of autonomic nervous system, as considered in the suggested works ^1^^,^^2^, have not been taken into account and that should be added at least as limitation.

I have read this submission. I believe that I have an appropriate level of expertise to confirm that it is of an acceptable scientific standard, however I have significant reservations, as outlined above.

## References

[1] BariVRanucciMDe MariaBCairoBPistuddiVPortaA: Model-based directional analysis of cardiovascular variability identifies patients developing atrial fibrillation after coronary artery bypass grafting. *Int J Cardiol*.2018;258: 10.1016/j.ijcard.2018.01.071 97-102 10.1016/j.ijcard.2018.01.071 29426634

[2] HogueCWDomitrovichPPSteinPKDespotisGDReLSchuesslerRBKleigerRERottmanJN: RR interval dynamics before atrial fibrillation in patients after coronary artery bypass graft surgery. *Circulation*.1998;98(5) :429-34 971409310.1161/01.cir.98.5.429

# Response

**Published:** 2018-07-02

**Authors:** Ahmad Farouk Musa

**Affiliations:** Monash University Malaysia, Malaysia

Authors propose a retrospective study about the incidence of post operative atrial fibrillation (POAF) in patients undergoing coronary artery bypass surgery in Malaysia.

To do so, 637 medical records have been considered, including several confounding factors that could be determinant of the outcome.

Results show that the incidence of POAF in Malaysia is similar to the European one.

The paper is well written and factors are correctly described.

Methods and statistics are appropriate.

I only have some concerns:

- The paper is quite long and it could be considered to try to revise some parts. I would like to suggest the authors try to revise some paragraphs, discarding the less necessary and focusing on the more important information, in order to improve readability.

 

**Response**: Thank you for your constructive comments. I have revised the manuscript and made it more readable.

 

- To strengthen the association with predictive factors of POAF it would be interesting to see also ROC curve showing the value of the area under the curve. That could result from the multivariate logistic regression analysis determined by including all factors resulting predictive for POAF.

 

**Response**:  We employed three methods to assess the goodness-of-fit of the regression model to the data: Hosmer-Lemeshow test, Classification Table, and the Area Under the RIC Curve (AUC).

**Model Fit Assessment**

The Hosmer-Lemeshow test (below) indicated that the model is a good fit to the data ( *p*=0.99) since the *p* value exceeded 0.05.

**Hosmer and Lemeshow Test**

Step      Chi-square     df         Sig.

1            1.664              8        .990

Further, the contingency table for the Hosmer and Lemeshow test (below) also indicated that the expected/predicted counts of the model clearly shows small differences between the observed and expected counts, thereby confirming the goodness of model fit.

**Contingency Table for Hosmer and Lemeshow Test**

                                 POAF = 0                                POAF = 1

                       Observed        Expected          Observed      Expected         Total

Step 1   1               56            56.149                     7                6.851              63

              2               54            53.080                     9                9.920              62

              3               53            50.677                    10              12.323             63

              4               49            48.237                    14             14.763              63

              5               44            46.367                    19             16.633              63

              6               44            44.852                    19             18.148              63

              7                41           42.710                    22              20.290             63

              8                42            40.215                    21             22.785             63

              9                37            37.236                    26             25.764             63

             10               30            30.476                    35             34.524             65

Using the Classification Table (below), the table shows that the model correctly predicts POAF by more than 70% (71.4%), thereby indicating a good fit of the regression model to the data.

**Classification Table ^a^**                        

                                                                     Predicted

                                           

                                                     POAF                      Percentage

          Observed                      0               1                   Correct

Step 1    POAF   0                433            17                      96.2

                            1                164            18                        9.9

                Overall percentage                                            71.4

----------------------------------------------                                          

a. The cut value is .500

Finally, the Area Under the ROC Curve (AUC) (below) indicated that the AUC is 0.663, which is slightly below the cut-off level for goodness-of-fit (70%) with a 95% CI of between 0.617 to 0.709. Examining the lower level of the 95%CI reveals that it is greater than the cut-off level of 0.5, thereby confirming the good fit of the regression model to the data.

**Area Under the Curve**

Test Result Variable(s):   Predicted probability 

^^Asymptotic 95% Confidence Interval

  Area    Std. Error ^a      ^Asymptotic Sig. ^b ^     Lower Bound    Upper Bound

.663            .023             .000                          .617                .709

The test result variable(s): Predicted probability has at least one tie between the positive actual state group and the negative actual state group. Statistics may be biased.

a. Under the nonparametric assumption

b. Null hypothesis: true area = 0.5

The AUC is graphically illustrated in the figure here.

 

- Some possible predictive factors, as the state of autonomic nervous system, as considered in the suggested works ^1^ ^,^ ^2^, have not been taken into account and that should be added at least as limitation.

 

**Response:** Thanks for pointing this our Professor. Yes, we have omitted the autonomic nervous system as independent predictors of AF post-CABG since this is a retrospective review and we did not monitor such changes in our patients. This will be noted in the Limitation of the study.

# Peer review: Referee response for version 1

**DOI:** 10.5256/f1000research.14368.r34013

**Published:** 2018-05-29

**Authors:** Johannes Steindl

**Affiliations:** 1 Department of Cardiac Surgery, Paracelsus Medical University, Salzburg, Austria

Postoperative atrial fibrillation after (POAF) is a common complication after coronary artery bypass grafting (CABG) and cardiac surgery in general.

 In this article Musa and colleagues perform a retrospective, single-center analysis of 637 patients undergoing CABG in order to identify the incidence of POAF, its associated morbidity and mortality as well as its pre- and intraoperative risk factors. The authors compare their results to previous studies and also point out their regional relevance by comparison between Malaysia’s major ethnic groups.

 From a technical point of view, the paper is well written. The Introduction section includes a very detailed literature review summarizing available data about incidence, mortality and risk factors of POAF after CABG. This part of the article indicates the complexity of POAF with a high amount of variables possibly influencing its aetiology and pathogenesis. The Methods section describes the study design and sample size calculation, the inclusion and exclusion criteria are plausible and the statistical methods are well chosen. In the Results and Discussion section, a detailed analysis of the collected data is performed, comparing their own results to previous studies and literature. Finally, the Conclusion section includes a short recap and a future perspective for further studies.

 In order to give a helpful feedback to the authors I want to divide my review in 2 sections: “technical aspects” and “intended relevance of the paper”.

 Technical aspects:

**Introduction:**

- I want to point out that the Introduction offers an excellent insight into POAF literature and the current state of research on this topic.

 

**Methods:**

- Page 4: The sample size was calculated in order to “investigate the outcome”. Please give a more detailed definition for the “outcome” you wanted to analyze. Which parameters did you look for in the first place (i.e. incidence, mortality, etc.)? Was the sample size large enough for all your endpoints? 
- The inclusion/exclusion criteria as well as the statistical methods were chosen appropriately.

 

**Results:**

- To provide a clear design of the tables I would recommend to include the total number of patients (637), the number of Non-POAF patients (454) and POAF patients (183) into the tables, since some of the percentage numbers are a little bit confusing at first sight. So, for example, you may mention those numbers in the table’s head line or within each field (i.e. Table 1: Male gender 521/637 (81.8). 
- Page 5, Table 2: “Time from surgery to POAF” should be corrected to “hours” instead of “minutes”
- Page 7, Operative details: The section about on-pump vs. off-pump CABG could be written a bit more understandable (i.e. the percentage of POAF in the on-pump group vs. the percentage of POAF in the off-pump group).
- Page 8, Postoperative stay: If available, the cause of prolonged stay on ICU and HDU (i.e. hemodynamic instability, rhythm control, non cardiac reasons) would be very interesting to know, in order to determine if there might be a causal relationship between POAF and the prolonged ICU stay or not.
- Data about postoperative cardiac pacing and its influence on POAF would be very interesting. What is your protocol of postoperative atrial/ventricular pacing? 
- Did you collect any data about the completeness of revascularization and its influence on POAF?  

 

**Discussion:**

- Page 11: Please explain why you selected the 48-hour timeframe to identify patients for prophylactic anti-coagulation. What was the number of patients with a POAF episode of > 24 hours? What is your protocol for recurring POAF? Do you start anticoagulation in those patients? (and maybe for the discussion section: what do you think is a good follow up protocol for those patients?) 

**Conclusion: **

- What are your thoughts about the importance of the difference of POAF incidence between the different ethical groups? Do you think there is any clinical relevance? Is it an interesting fact for research about genetic influences on POAF?
- Please explain more detailed what surgical methods you think about, that could be improved? Bicaval cannulation (as you mentioned) is hardly done in isolated CABG. The reduction of cross clamp time is probably the goal of every surgical procedure. So in my opinion the current possibilities for surgical improvement are rather limited.
- Would it be possible to estimate the additional costs caused by POAF per year in your national health care system?

 

**Relevance of the paper:**

 I think it is important to determine the target audience for this paper as well the article’s intended impact. If the author’s main intention is to present an analysis of POAF by using a local patient cohort, the paper is solidly written. However, despite the analysis of differences between Malayan ethnical groups, the novelty and scientific relevance of the information presented is limited.

 Although my limited knowledge about the Malayan health care system and its medical research institutions, in my opinion this article has huge potential to emphasize a discussion about improvement of the national research and data collection system. The authors mention on page 4 that there is currently no data available about the total number of CABG performed in Malaysia per year. Such information could be collected through manageable financial efforts by installing a national database for cardiac surgery, comparable to already existing databases in Europe or the United States. Such a database would profoundly facilitate further scientific research as well as quality control. Furthermore the author’s mention that prospective studies have to be performed in future. One of the main problems might be that the costs for that kind of studies often are enormous and that there might be a lack of industrial sponsors. If the authors were able to determine an estimation of the real-world additional costs of POAF for the the national health care budget, this might facilitate national sponsorship for research targeting to reduce these costs.

 However, in summary I support the indexing of the paper in regard of the revisions mentioned above.

I have read this submission. I believe that I have an appropriate level of expertise to confirm that it is of an acceptable scientific standard.

# Response

**Published:** 2018-07-02

**Authors:** Ahmad Farouk Musa

**Affiliations:** Monash University Malaysia, Malaysia

Postoperative atrial fibrillation after (POAF) is a common complication after coronary artery bypass grafting (CABG) and cardiac surgery in general.

In this article Musa and colleagues perform a retrospective, single-center analysis of 637 patients undergoing CABG in order to identify the incidence of POAF, its associated morbidity and mortality as well as its pre- and intraoperative risk factors. The authors compare their results to previous studies and also point out their regional relevance by comparison between Malaysia’s major ethnic groups.

From a technical point of view, the paper is well written. The Introduction section includes a very detailed literature review summarizing available data about incidence, mortality and risk factors of POAF after CABG. This part of the article indicates the complexity of POAF with a high amount of variables possibly influencing its aetiology and pathogenesis. The Methods section describes the study design and sample size calculation, the inclusion and exclusion criteria are plausible and the statistical methods are well chosen. In the Results and Discussion section, a detailed analysis of the collected data is performed, comparing their own results to previous studies and literature. Finally, the Conclusion section includes a short recap and a future perspective for further studies.

In order to give a helpful feedback to the authors I want to divide my review in 2 sections: “technical aspects” and “intended relevance of the paper”.

Technical aspects:

**Introduction:**

- I want to point out that the Introduction offers an excellent insight into POAF literature and the current state of research on this topic.

 

**Response**: Thank you Professor.

**Methods:**

- Page 4: The sample size was calculated in order to “investigate the outcome”. Please give a more detailed definition for the “outcome” you wanted to analyze. Which parameters did you look for in the first place (i.e. incidence, mortality, etc.)? Was the sample size large enough for all your endpoints? 

 

**Response**: We have revised the sample size calculation as follows:

**Primary endpoint** is the incidence of POAF: Using the Raosoft® sample size calculator software ^28^, the calculated sample size was 377 (margin of error: 5%; confidence interval: 95%; estimated population size: 20,000; and response distribution: 50%). This is the minimum recommended sample size for the study. However, using the formula for sample size calculation based on the incidence of POAF of 30% ^9,10,12,13^, the estimated sample size (n) = Z ^2^P(1-P)/d ^2^, where Z= Z statistic for a level of confidence, P=expected prevalence or proportion, and d=precision (Ref: Naing et al 2006). Therefore, n = [(1.96)2*0.3*0.7]/(0.05) ^2^ = 323. Taking into account a possible 20% attrition rate, the minimum sample size required to determine the incidence of POAF = 323 + (0.2*323) = 388.

**Secondary endpoints** are the mortality and morbidity rates, duration of ICU, HDU, and hospital stay. Sample size calculations for these endpoints are as follows (Omer et al 2016):

- **Mortality rate (1.9%): n=** Z ^2^P(1-P)/d ^2^ = [(1.96) ^2^*0.019*0.981]/(0.05) ^2^ = **28.6 approx. = 29**
- **Length of Hospital Stay (LOHS): **Using the P&S Software (Dupont & Plummer 1990), we calculate the sample size for a reported LOHS of mean 12.7 +6.6 days for patients with POAF and mean of 10.3 +8.9 days for those without (Omer et al 2016), as follows: **α=0.05, 1-β=0.80, δ=2.4, σ=0.641, m=1. Therefore, n=4**

 

Based on the above sample size calculations, we will adopt the highest sample size calculated, i.e., n=388 as the minimum sample size required for this study as this sample size is adequate for both primary and secondary endpoints.

**References:**

Dupont WD, Plummer WD: "Power and Sample Size Calculations: A Review and Computer Program", Controlled Clinical Trials 1990; 11:116-28.

Naing L, Winn T, Rusli BN: Practical issues in calculating the sample size for prevalence studies. *Archives of Orofacial Sciences* 2006; (1):9-14.

Omer S, Cornwell LD, Bakshi A et al: Incidence, predictors, and impact of postoperative atrial fibrillation after coronary artery bypass grafting in military veterans 2016; 43(5):397-403

 

- The inclusion/exclusion criteria as well as the statistical methods were chosen appropriately.

 

**Response**: Thank you.

**Results:**

- To provide a clear design of the tables I would recommend to include the total number of patients (637), the number of Non-POAF patients (454) and POAF patients (183) into the tables, since some of the percentage numbers are a little bit confusing at first sight. So, for example, you may mention those numbers in the table’s head line or within each field (i.e. Table 1: Male gender 521/637 (81.8). 

 

**Response**: Thank you for pointing this out. I have made the necessary corrections.

 

- Page 5, Table 2: “Time from surgery to POAF” should be corrected to “hours” instead of “minutes”

 

**Response: **Thank you for pointing out the mistake. I have changed it.

 

- Page 7, Operative details: The section about on-pump vs. off-pump CABG could be written a bit more understandable (i.e. the percentage of POAF in the on-pump group vs. the percentage of POAF in the off-pump group).

 

**Response: **Thank you. I have done the necessary correction to make it more clearer. We noticed that 29.3% of on-pump patients developed AF post-operatively as compared to 18.2% in the off-pimp cases. However the difference is not statistically significant.

 

- Page 8, Postoperative stay: If available, the cause of prolonged stay on ICU and HDU (i.e. hemodynamic instability, rhythm control, non-cardiac reasons) would be very interesting to know, in order to determine if there might be a causal relationship between POAF and the prolonged ICU stay or not.

 

**Response**: Since this is a retrospective review, we have not really looked into the related factors apart from POAF and the treatment to bring the patient back to sinus rhythm. However we would be interested to look into the causal relationship between POAF and the prolonged ICU stay in these subsets of patients.

 

- Data about postoperative cardiac pacing and its influence on POAF would be very interesting. What is your protocol of postoperative atrial/ventricular pacing? D in a future study to look in

 

**Response:** We routinely placed prophylactic right ventricle temporary epicardial pacing wire. We don’t normally place atrial pacing wire. And our patients are not routinely paced post-operatively.

 

- Did you collect any data about the completeness of revascularization and its influence on POAF?  

 

**Response:** Unfortunately we do not have any data nor conduct any study intra- or post-operatively to determine the completeness of revascularization. We do not use Flowmeter during surgery to measure the blood flow in the graft though this idea is exciting.

**Discussion:**

- Page 11: Please explain why you selected the 48-hour timeframe to identify patients for prophylactic anti-coagulation. What was the number of patients with a POAF episode of > 24 hours? What is your protocol for recurring POAF? Do you start anticoagulation in those patients? (and maybe for the discussion section: what do you think is a good follow up protocol for those patients?) 

 

**Response:** We noticed that there is a bleeding tendency in our population as compared to Western patients. And we hardly counter any thromboembolic patients in the first 48 hours. So the benefit of anticoagulation doesn’t outweigh the risk of bleeding in the first 48-hours.

And if the POAF recurs after the first 48hours, patients will be started on LMWH overlapping oral Warfarin. Patients will be given Warfarin for the first 6-weeks with a target INR of 2.0-3.0. The patients will be followed up at six-weeks and if found to be in sinus rhythm, the Warfarin will be taken off.

**Conclusion:**

- What are your thoughts about the importance of the difference of POAF incidence between the different ethical groups? Do you think there is any clinical relevance? Is it an interesting fact for research about genetic influences on POAF?

 

**Response:** Our study showed a significantly lower incidence of POAF in the Indian community., a finding that was found in a similar study in Singapore. In fact, Indians in general are found to have a lower incidence of AF. Epidemiological studies conducted across multiple nations showed this trend previously. This shows that Indians are more resistant to develop AF in general, not just post-operative AF. Hence we think that genetic factor that confers immunity relatively to the Indian community could not be ruled out.

It will be very interesting to investigate if the pre-op preventive strategies could significantly reduce the development of POAF in the Indian community. And if they do, will the benefit outweigh the risk. The number needed to treat will definitely be higher in the Indian community. So the cost-benefit analysis may not be generalised to this group of people.

 

- Please explain more detailed what surgical methods you think about, that could be improved? Bicaval cannulation (as you mentioned) is hardly done in isolated CABG. The reduction of cross clamp time is probably the goal of every surgical procedure. So in my opinion the current possibilities for surgical improvement are rather limited.

 

**Response:** Yes Professor, the possibilities of improvement is rather limited. But we feel that despite the advancement in cardiothoracic surgery, perhaps using retrograde cardioplegia together with the routine antegrade has the potential to minimize the occurrence of POAF. Studies have shown that giving both antegrade and retrograde cardioplegia provides better myocardial protection that either technique alone – in our centre, the antegrade – and ensures good cardioplegic distribution to the left and right ventricle, and allows regional delivery of cardioplegic flow to region supplied by the occluded arteries. Though we have to admit that this was just our postulation based on our anecdotal experience in Melbourne, Australia. We have yet to come across any studies that suggest such a correlation.

 

- Would it be possible to estimate the additional costs caused by POAF per year in your national health care system?

 

**Response:** Yes Professor. I believe this is possible but would definitely require a thorough study. We have done a study many years ago in looking at the cost-effectiveness of performing endoscopic vein harvesting to that of leg wound management and dressing in open saphenous vein harvesting. So looking at the additional cost in managing POAF patients is not impossible but would require time, effort, and full cooperation by the Institution. 

**Relevance of the paper:**

I think it is important to determine the target audience for this paper as well the article’s intended impact. If the author’s main intention is to present an analysis of POAF by using a local patient cohort, the paper is solidly written. However, despite the analysis of differences between Malayan ethnical groups, the novelty and scientific relevance of the information presented is limited.

Although my limited knowledge about the Malayan health care system and its medical research institutions, in my opinion this article has huge potential to emphasize a discussion about improvement of the national research and data collection system. The authors mention on page 4 that there is currently no data available about the total number of CABG performed in Malaysia per year. Such information could be collected through manageable financial efforts by installing a national database for cardiac surgery, comparable to already existing databases in Europe or the United States. Such a database would profoundly facilitate further scientific research as well as quality control. Furthermore the author’s mention that prospective studies have to be performed in future. One of the main problems might be that the costs for that kind of studies often are enormous and that there might be a lack of industrial sponsors. If the authors were able to determine an estimation of the real-world additional costs of POAF for the the national health care budget, this might facilitate national sponsorship for research targeting to reduce these costs.

However, in summary I support the indexing of the paper in regard of the revisions mentioned above.

**Response:** Thanks so much Professor for your advice and support. We definitely will take this matter up to the higher authorities.
